# Growth kinetics of Cu_6_Sn_5_ intermetallic compound at liquid-solid interfaces in Cu/Sn/Cu interconnects under temperature gradient

**DOI:** 10.1038/srep13491

**Published:** 2015-08-27

**Authors:** N. Zhao, Y. Zhong, M.L. Huang, H.T. Ma, W. Dong

**Affiliations:** 1 School of Materials Science and Engineering, Dalian University of Technology, Dalian 116024, China

## Abstract

The growth behavior of intermetallic compounds (IMCs) at the liquid-solid interfaces in Cu/Sn/Cu interconnects during reflow at 250 °C and 280 °C on a hot plate was investigated. Being different from the symmetrical growth during isothermal aging, the interfacial IMCs showed clearly asymmetrical growth during reflow, i.e., the growth of Cu_6_Sn_5_ IMC at the cold end was significantly enhanced while that of Cu_3_Sn IMC was hindered especially at the hot end. It was found that the temperature gradient had caused the mass migration of Cu atoms from the hot end toward the cold end, resulting in sufficient Cu atomic flux for interfacial reaction at the cold end while inadequate Cu atomic flux at the hot end. The growth mechanism was considered as reaction/thermomigration-controlled at the cold end and grain boundary diffusion/thermomigration-controlled at the hot end. A growth model was established to explain the growth kinetics of the Cu_6_Sn_5_ IMC at both cold and hot ends. The molar heat of transport of Cu atoms in molten Sn was calculated as + 11.12 kJ/mol at 250 °C and + 14.65 kJ/mol at 280 °C. The corresponding driving force of thermomigration in molten Sn was estimated as 4.82 × 10^−19^ N and 6.80 × 10^−19^ N.

In electronic packaging technology, the formation of interfacial intermetallic compound (IMC) during soldering reaction is essential to realize a reliable metallurgy interconnection between solder and under bump metallizations (UBMs). However, the brittle nature of IMC makes its thickness and morphology must be effectively controlled^1^. As electronic products are continuously pursuing high-performance, multi-function and miniaturization, the micro bumps (μ-bumps) for chip interconnections in three dimensional integrated circuit (3D IC) packaging are an order of magnitude smaller in size than the solder joints in flip chip packaging. The shrinking of interconnection results in a significant increase in the volume proportion of interfacial IMC to the whole solder joint^2^,^3^,^4^. The reliability of micro solder joints becomes more and more sensitive to the growth of interfacial IMC.

Thermomigration (TM) is one of the simultaneous heat and mass transfer phenomena that occurs in a mixture, as a result of an external temperature gradient imposed across the mixture. Under a certain temperature gradient, TM may occur in solid solder joints^5^,^6^,^7^,^8^. It was found that Cu atoms quickly diffused from Cu UBM into Sn matrix at the hot/chip end of Cu/Sn-3.5Ag/Cu flip chip solder joint under a temperature gradient of 1143 °C/cm at 150 °C^6^. Under a temperature gradient of 7308 °C/cm in Sn-2.5Ag micro bumps at 145 °C, Ni atoms were driven to migrate toward the cold end, as evidenced by a faster Ni_3_Sn_4_ growth at the cold end and a more apparent Ni consumption at the hot end^8^. Since the interdiffusion of atoms between solder and UBMs markedly affects interfacial reaction, temperature gradient that enhances the directional diffusion of metal atoms and induces the redistribution of elements will significantly influence the growth behavior of interfacial IMC.

Solder joints/bumps commonly undergo several reflows in the process of flip chip and 3D IC technologies^9^. There may exist a temperature gradient during reflow in oven or on hot plate due to the difference in thermal conductivities of chip, substrate and solder alloy. In addition, heat is applied through one of the chips for hot compression method (thermo-compression bonding) and temperature gradient across the micro bumps will be generated^5^,^10^. Since the diffusivity of atoms in liquid solder is significantly larger than that in solid solder, a small temperature gradient may induce a mass migration of atoms. Thus, the growth of interfacial IMC becomes more sensitive to temperature difference in solder joints during soldering process. Guo *et al.*^11^ found that the interfacial Cu_6_Sn_5_ was much thicker at the cold end whereas the consumption of Cu was much faster at the hot end in Cu/Sn-2.5Ag/Cu solder joints during reflow at 260 °C on a hot plate, due to the rapid migration of Cu atoms under a simulated temperature gradient of 51 °C/cm. Qu *et al.*^12^ *in situ* studied the soldering interfacial reactions under a temperature gradient of 82.2 °C/cm at 350 °C using synchrotron radiation real-time imaging technology, and asymmetrical growth and morphology of interfacial IMCs between the cold and hot ends were clearly observed.

So far, the growth kinetics of interfacial IMC under temperature gradient during a soldering process is still unknown to us. The effect of TM on interfacial IMC growth needs an in-depth study. In the present work, the diffusion behavior of Cu atoms and its effect on liquid-solid reactions in Cu/Sn/Cu interconnects undergoing TM were investigated. The growth kinetics of interfacial Cu_6_Sn_5_ IMC at both hot and cold ends was identified.

## Results

### Interfacial IMC growth during isothermal aging

**Figure 1:**
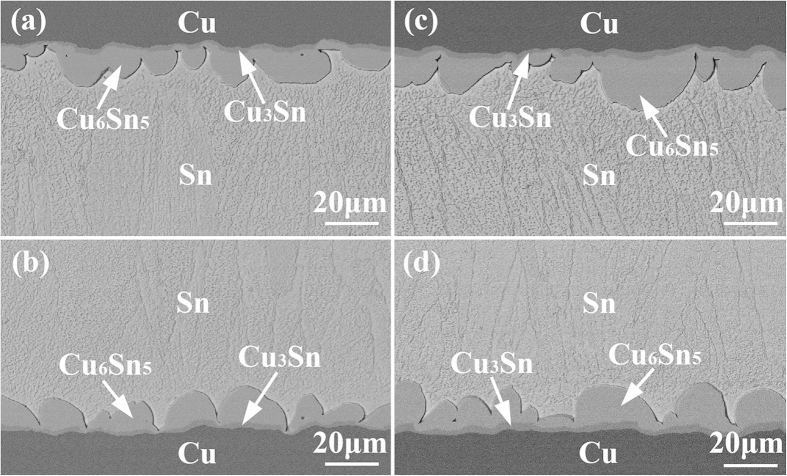
Cross-sectional microstructure of the Cu/Sn/Cu interconnects after isothermal aging for 120 min: (a,b) 250 °C; (c,d) 280 °C.

For the as-soldered state, a continuous Cu_6_Sn_5_ IMC layer with an average thickness of 0.32 μm was observed at each interface of the Cu/Sn/Cu interconnects. Such a thin initial interfacial IMC would have little influence on the subsequent interfacial reactions. Figure 1 shows the cross-sectional microstructure of the Cu/Sn/Cu interconnects after isothermal aging for 120 min. When aged at 250 °C, bilayer IMCs with thick scallop-like Cu_6_Sn_5_ of 8.45 μm close to the Sn matrix and thin Cu_3_Sn of 1.53 μm close to the Cu substrate formed at both interfaces. When aged at 280 °C, the interfacial Cu_6_Sn_5_ and Cu_3_Sn IMCs grew to 10.17 μm and 2.34 μm, respectively. It is noted that the growth of the IMCs at the two interfaces was symmetrical undergoing isothermal aging.

### Interfacial IMC growth under temperature gradient

**Figure 2:**
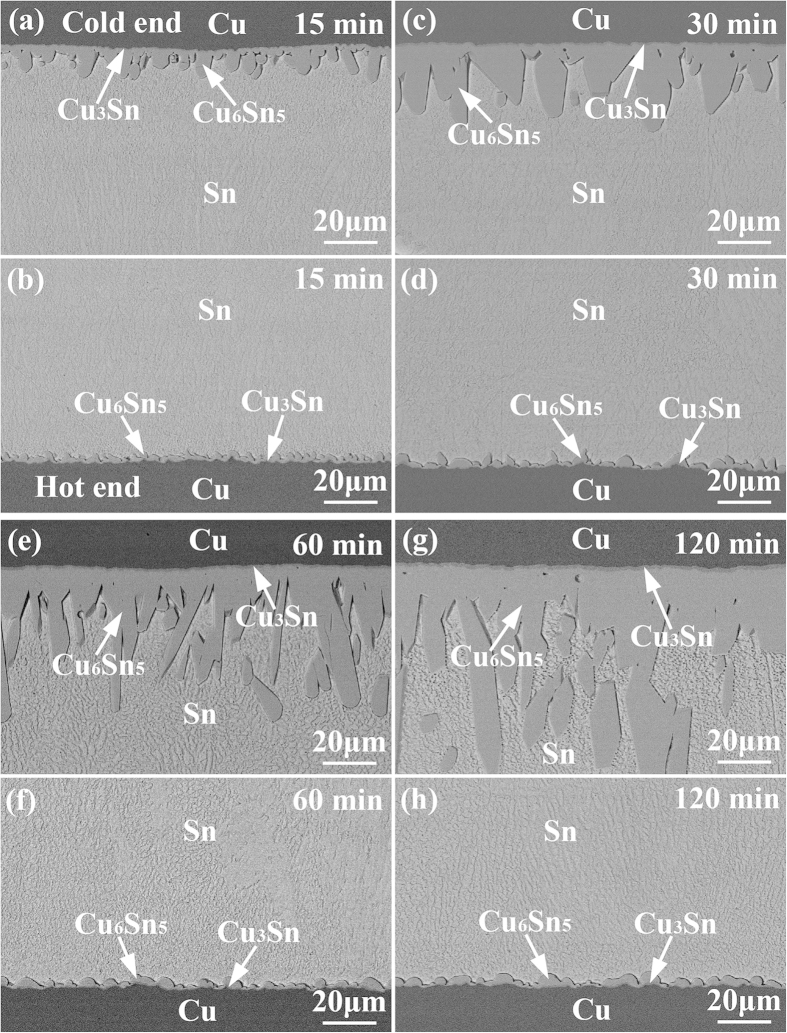
Microstructural evolution of the Cu/Sn/Cu solder joints after reflowed at 250 °C for different durations: (a,c,e and g) cold end, and (b,d,f and h) hot end.

**Figure 3:**
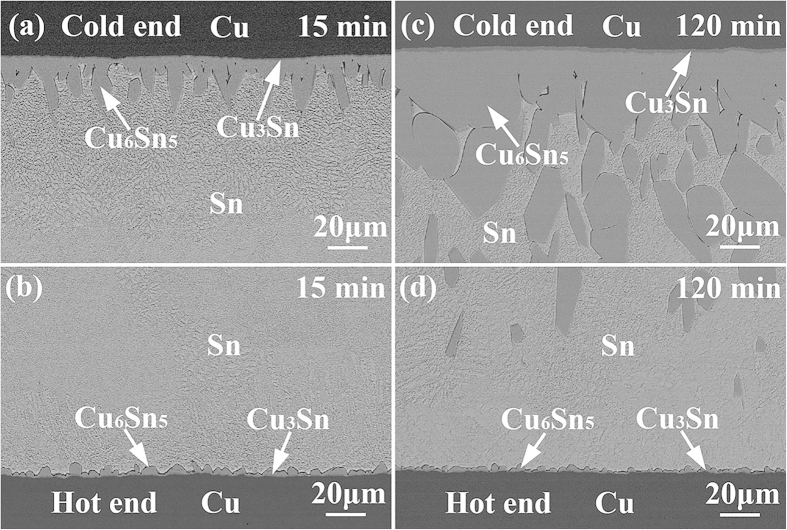
Microstructural evolution of the Cu/Sn/Cu solder joints after reflowed at 280 °C for 30 min and 120 min: (a,c) cold side, and (b,d) hot side.

Figure 2 shows the microstructural evolution of the Cu/Sn/Cu interconnects after reflow at 250 °C for different durations on a hot plate. The bottom interfaces closer to the hot plate were the hot ends, whereas the top interfaces were the cold ends. The interfacial IMCs at both ends remained Cu_6_Sn_5_ and Cu_3_Sn. However, asymmetrical growth of the interfacial IMCs was clearly observed. The Cu_6_Sn_5_ at the cold end grew much faster than that at the hot end, while the growth of the Cu_3_Sn was hindered, especially at the hot end. Figure 3 presents the microstructural evolution of the Cu/Sn/Cu interconnects after reflow at 280 °C for different durations on a hot plate. The asymmetrical growth of the interfacial IMCs between the hot and cold ends became more obvious. In addition, some Cu_6_Sn_5_ IMCs spalled from the cold end after reflow for 60 min, as shown in Figs 2 and 3. Since Cu_6_Sn_5_ is quite brittle, fracture of the long prismatic Cu_6_Sn_5_ IMCs could occur when they suffered concentration and temperature fluctuations during cooling as evidenced by the serrated boundaries toward the cold end.

Since no external field, such as electric or stress, was introduced during reflow, temperature gradient between the hot and cold ends was responsible for the asymmetrical IMC growth. Table 1 shows the concentration of Cu across the solder layer after solidification. The highest Cu concentration always existed in the middle of the solder layer rather than near the cold or hot end. However, due to the continuity of Cu concentration in liquid Sn, it is deduced that the concentration gradient of Cu was from the hot end to the cold end during reflow, i.e., the highest Cu concentration driven by temperature gradient should exist at the cold end before cooling. Since the precipitation of interfacial Cu_6_Sn_5_ was quite fast during cooling^12^, the Cu concentration near the interfaces could markedly reduce before solidification, especially at the cold end. As a result, the Cu concentrations near the interfaces became lower than that in the middle of the solder layer after solidification. Similar concentration distribution of the dominated TM species was also found in solid state solder joints during TM^7^,^13^. Furthermore, the steady state solute concentration gradient is related to the impressed temperature gradient through the expression^14^


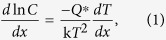


where *C* is the solute (Cu) concentration, Q* is the heat of transport, k is the Boltzmann’s constant, and *T* is the absolute temperature.

As Cu atoms were continuously driven to migrate from the hot end to the cold end by temperature gradient, Q* was positive. According to equation (1), the Cu concentration gradient and the temperature gradient were in opposite directions, i.e., the Cu concentration at the cold end was higher than that at the hot end. As a result, the interfacial IMC growth at the cold end was promoted due to the larger amount and size of Cu-Sn clusters^15^,^16^, while that at the hot end was inhibited. The thick interfacial IMC layer and the high Cu concentration both restrained the dissolution of the Cu substrate at the cold end. On the contrary, the thin interfacial IMC layer and the low Cu concentration both promoted the dissolution of the Cu substrate at the hot end, as evidenced by the rough IMC/Cu interfaces shown in Figs 2 and 3. Thus, the fast IMC growth at the cold end was mainly sustained by the TM of Cu from the hot end. TM of atoms in liquid solder plays a significant effect on interfacial IMC growth.

### Simulation of temperature gradient

During reflow on hot plate, the main consideration of heat transfer was air convection and heat radiation, since Cu has a high thermal conductivity (401 W/m·K). The rate equation for convective heat transfer can be expressed by the Newton rate equation


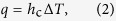


where *q* is the rate of convective heat transfer, ∆*T* is the temperature difference between surface and fluid, and *h*_c_ is the convective heat transfer coefficient.

The surface of the interconnects was considered as an ideal blackbody with gray surface that was completely surrounded. The radiation heat transfer coefficient *h*_r_ can be expressed as


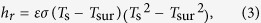


where *ε* is the radiative property of the surface termed emissivity, *σ* is the Stefan-Boltzmann constant, and *T*_s_ and *T*_sur_ are the surface temperatures of the interconnects and the walls of the room, respectively.

**Figure 4:**
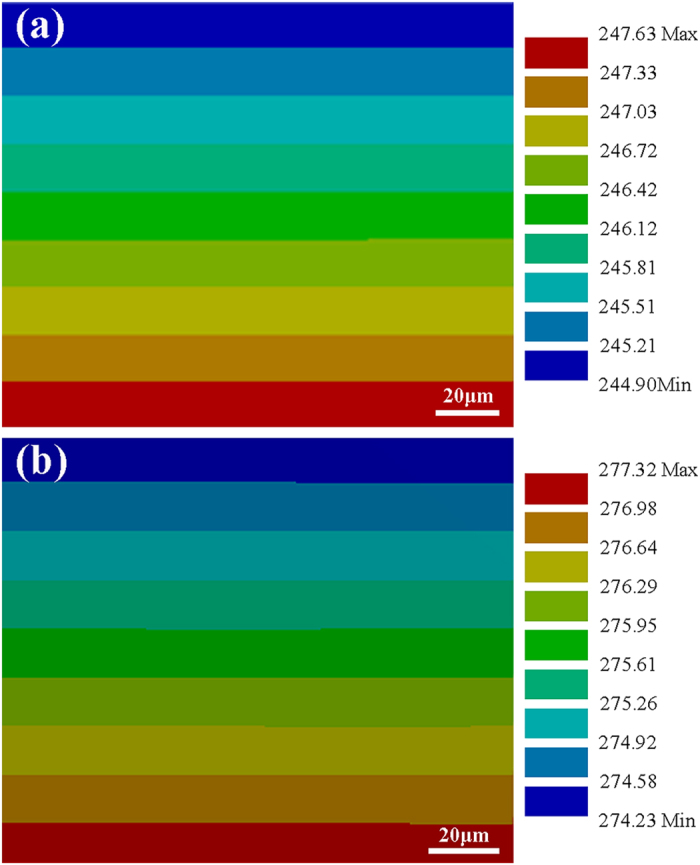
Simulated temperature distribution in the solder layer during reflow at (a) 250 °C and (b) 280 °C.

The combined heat transfer coefficient of convection and radiation *h*_t_ = *h*_c_ + *h*_r_. Figure 4 shows the simulated temperature distribution in the liquid solder layers during reflow using FEM when *h*_t_ was set to 65 W/m^2^·K. The simulated temperatures of the top regions of the interconnects were 235.16 °C and 263.21 °C when reflowed at 250 °C and 280 °C, respectively, which well agreed with the measured values by thermocouple. Correspondingly, the temperature gradients across the solder layers were 136.50 °C/cm and 154.50 °C/cm.

## Discussion

### Growth kinetics of interfacial Cu_6_Sn_5_

The IMC growth at a solder/Cu interface can be adequately modeled with the empirical power low as follows


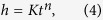


where *h* is the thickness of interfacial IMC, *K* is the coefficient of IMC growth rate, *t* is the reaction time and *n* is the time exponent. When *n* = 1/3, the interfacial IMC growth follows the parabolic law and is grain boundary diffusion-controlled^17^; when *n* = 1/2, the interfacial IMC growth follows the parabolic law and is volume diffusion-controlled^18^; when *n* = 1, the interfacial IMC growth follows the linear law and is reaction-controlled^19^.

It can derive from equation (4) that


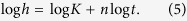


Therefore, log *h* shows a linear relationship with log*t*. Since Cu_6_Sn_5_ was the dominant reaction product at all the interfaces, the growth kinetics of Cu_6_Sn_5_ was discussed.

**Figure 5:**
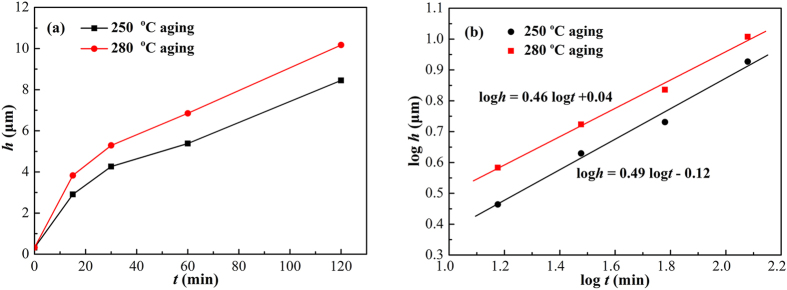
Thickness of the interfacial Cu_6_Sn_5_ as a function of aging time: (a) *h*–*t*; (b) log*h*–log*t*.

Figure 5 shows the thickness of interfacial Cu_6_Sn_5_ as a function of aging time. As shown in Fig. 5(a), the interfacial Cu_6_Sn_5_ grew parabolically with aging time, indicating a diffusion-controlled growth mechanism. As shown in Fig. 5(b), the thickness of interfacial Cu_6_Sn_5_ increased linearly with aging time in log-log format. The values of *n* after linear fitting were 0.44 and 0.45 at 250 °C and 280 °C, respectively. Thus, the growth of interfacial Cu_6_Sn_5_ during isothermal aging was controlled by the volume diffusion of Cu atoms to the Sn/Cu interfaces, which agreed well with the results of Li *et al.*^18^.

**Figure 6:**
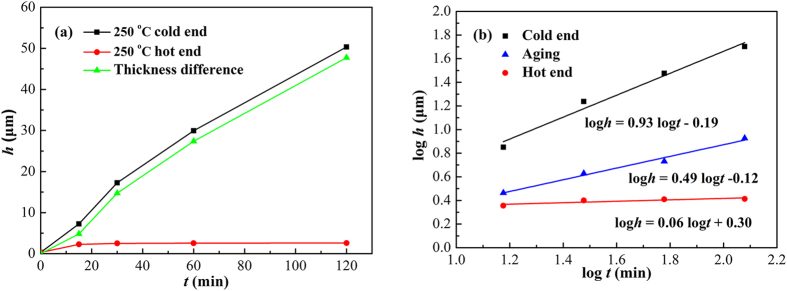
Thickness of interfacial Cu_6_Sn_5_ as a function of TM time at 250 °C: (a) *h*–*t*; (b) log*h*–log*t*.

Figure 6 shows the thickness of interfacial Cu_6_Sn_5_ as a function of TM time at 250 °C. As shown in Fig. 6(a), the Cu_6_Sn_5_ at the two interfaces presented distinct growth behavior. The Cu_6_Sn_5_ at the cold end grew rapidly and linearly with TM time, whereas that at the hot end grew relatively slow, resulting in an increasing difference in IMC thickness between the cold and hot ends undergoing TM. After TM for 120 min, the thicknesses of Cu_6_Sn_5_ at the cold and hot ends were 50.34 μm and 2.59 μm, respectively. As shown in Fig. 6(b), the thickness of Cu_6_Sn_5_ showed a good linear relationship with TM time in log-log format. The *n* for the cold end was 0.93, indicating a reaction-controlled growth mechanism; while the *n* for the hot end was 0.06, indicating that the interfacial Cu_6_Sn_5_ hardly grew. The data under isothermal aging at 250 °C were also plotted in Fig. 6(b) for comparison. It is noted that the growth rate of interfacial Cu_6_Sn_5_ under TM was much higher than that under isothermal aging.

**Figure 7:**
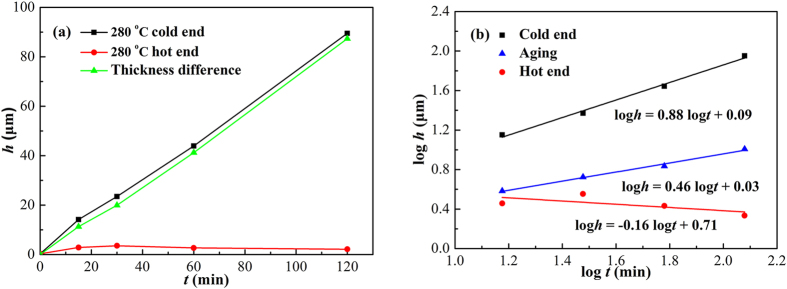
Thickness of interfacial Cu_6_Sn_5_ as a function of TM time at 280 °C: (a) *h*–*t*; (b) log*h*–log*t*.

Figure 7 shows the thickness of interfacial Cu_6_Sn_5_ as a function of TM time at 280 °C. The asymmetrical IMC growth between the hot and cold ends became more apparent comparing with that at 250 °C. The Cu_6_Sn_5_ at the cold end remained a linear growth with TM time, while that at the hot end turned to thinning after TM for 30 min. The *n* for the cold end was 0.88, indicating a reaction-controlled growth mechanism; while the *n* for the hot end was −0.16, indicating that the dissolution of interfacial Cu_6_Sn_5_ occurred. The *K* and *n* values under different reaction conditions were listed in Table 2.

During isothermal aging, though the interfacial IMCs grew faster at 280 °C than at 250 °C, their thickness different was small (less than 2 μm), as shown in Fig. 5(a). However, the thickness different between the interfacial IMCs at the cold end during reflow at 280 °C and 250 °C was obviously larger (even 40 μm), as shown in Figs 6 and 7. It can conclude that temperature gradient dominated the IMC growth rate during reflow, whereas reflow temperature contributed little.

The driving force of IMC formation is the difference of chemical potential between the two phases in contact. The IMC grows when the inward flux of the dominant diffusion species into IMC is larger than the outward flux, and decreases otherwise. Under TM, the growth kinetics of the interfacial Cu_6_Sn_5_ was discussed only considering Cu atomic flux, but neglecting Sn atomic flux, since Cu is the dominant diffusing species for the interfacial reaction in Cu/Sn system^20^. The Cu atomic flux 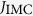 can be expressed as


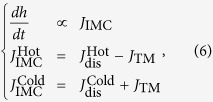


where 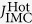 and 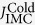 are the terms of 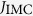 at the hot and cold ends, respectively, *J*_TM_ is the drift term of Cu in the liquid Sn due to thermomigration, and 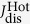 and 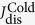 are the diffusion terms of Cu due to the dissolution of Cu substrates at the hot and cold ends, respectively.

**Figure 8:**
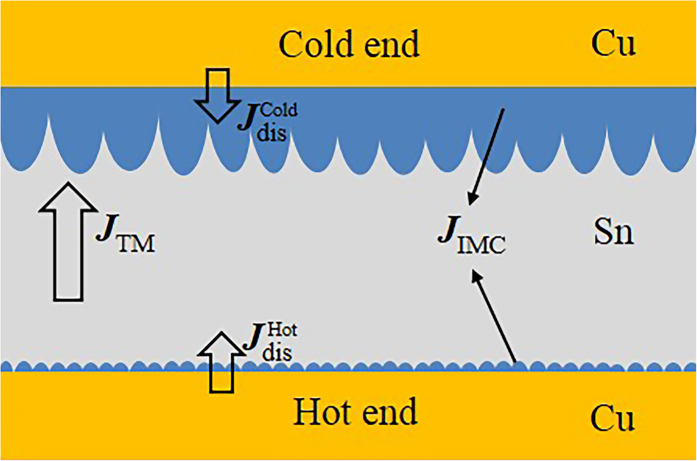
Schematic of atomic fluxes in the Cu/Sn/Cu interconnect.

Figure 8 shows the schematic of Cu atomic fluxes in the Cu/Sn/Cu interconnect under TM. At the cold end, the dissolution of the Cu substrate and the TM of Cu atoms from the hot end provided sufficient Cu atomic flux (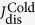 + 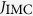) for interfacial IMC growth. It should note that as the direction of 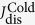 was against the temperature gradient, the IMC growth benefitted little from 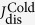 at the early stage of the reaction. With prolonged reaction, however, Cu atoms from the substrate could hardly diffuse across the thickening Cu_6_Sn_5_ layer against temperature gradient, resulting in a negligible 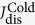. This was also proved by our previous *in situ* observations that the relative distance between the bubbles and the Cu substrate at the cold end remained unchanged, i.e., no dissolution of the Cu substrate occurred^12^. Consequently, the fast growth of the interfacial Cu_6_Sn_5_ at the cold end only sustained by *J*_TM_. We propose that the growth mechanism of the interfacial Cu_6_Sn_5_ at the cold end is reaction/thermomigration-controlled.

At the hot end, Cu atoms from the substrate could fast diffuse across the thin Cu_6_Sn_5_ layer by grain boundary diffusion along temperature gradient, resulting in a large 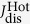. However, once dissolved into the liquid Sn, the Cu atoms migrated away immediately, leaving limited Cu atomic flux (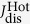
−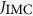) for interfacial IMC growth. Consequently, the growth mechanism of the interfacial Cu_6_Sn_5_ at the hot end was considered as grain boundary diffusion/thermomigration-controlled. When *J*_TM_ was comparable with 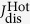, the interfacial Cu_6_Sn_5_ quitted growing, as shown in Figs 2 and 6 after TM for 15 min at 250 ^°^C. When *J*_TM_ exceeded 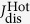, the dissolution of interfacial Cu_6_Sn_5_ occurred, as shown in Figs 3 and 7 after TM for 30 min at 280 ^°^C. Similar phenomenon of IMC decomposition was also observed at cathode interface in Cu/Sn/Cu interconnects undergoing eletromigration^21^.

It is also known that the growth and dissolution of interfacial IMC occur simultaneously during Sn/Cu liquid-solid reaction, as long as the liquid solder remains unsaturated with Cu^22^. According to Dybkov’s model^19^, the dissolution rate of interfacial IMC in liquid solder during reflow can be expressed as


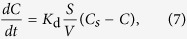


where *K*_d_ is a constant, *S* is the surface area of the IMCs in contact with the solder, *V* is the volume of the molten solder, and *C*s is the solubility of Cu in the molten solder at the reaction temperature. Therefore, the higher the *C*_s_-*C*, the higher the dissolution rate of interfacial IMC. At the cold end, since mass Cu atoms were driven to the interface by temperature gradient, the liquid solder was saturated or near-saturated with Cu, resulting in a low *C*_s_-*C*. The growth of interfacial IMC was dominant at the cold end. On the contrary, the liquid solder at the hot end was always unsaturated with Cu, resulting in a large *C*_s_-*C*. When the dissolution rate was over the growth rate, the interfacial Cu_6_Sn_5_ became thinner, as shown in Fig. 3(d).

### Heat of transport (Q*) of Cu in molten Sn

TM is the result of interaction between diffusion atoms and heat flow driven by the temperature gradient. The TM flux *J*_TM_ can be expressed as^5^


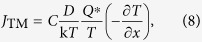


where *D* is the diffusion coefficient of Cu in the liquid solder, and ∂*T*/∂*x* is the temperature gradient. The heat of transport Q*, having the same dimension as chemical potential, is defined as the difference between heat energy carried by a moving atom per mole to the heat energy of atoms per mole at the hot end.

The driving force of thermomigration *F* is given as^5^


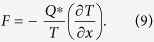


As mentioned above, the excessive growth of Cu_6_Sn_5_ IMC at the cold end is attribute to the TM of Cu atoms from the hot end. Thus, the atomic flux of Cu, 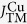, can be obtained by the variation in thickness of Cu_6_Sn_5_ at the cold end, namely the net growth of Cu_6_Sn_5_ from 60 min to 120 min. Therefore,


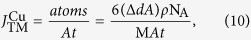


where *A* is the cross sectional area of the interconnect, *t* is the reaction time, N_A_ is the Avogadro’s number, Δ*d* is the increase of Cu_6_Sn_5_ thickness from 60 min to 120 min at the cold end, *ρ* is the density of Cu_6_Sn_5_ (8.28 g/cm^3^), and M is the molecular weight of Cu_6_Sn_5_.

According to equation (10), as Δ*d* is 20.39 μm at 250 °C and 45.55 μm at 280 °C, 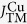 was calculated to be 1.74 × 10^16^ atoms/cm^2^s and 3.88 × 10^16^ atoms/cm^2^s, respectively. When TM at 250 °C, the temperature gradient is 136.50 °C/cm, the diffusivity of Cu in molten Sn is 3.16 × 10^−5^ cm^2^/s^23^ and the concentration (the solubility) of Cu in Sn is 1.25 wt.%^24^. Thus, according to equation (8), the molar heat of transport of Cu atoms in molten Sn at 250 °C was calculated as + 11.12 kJ/mol. Similarly, when TM at 280 °C, the temperature gradient is 154.50 °C/cm, the diffusivity of Cu in molten Sn is 3.93 × 10^−5^ cm^2^/s^23^, and the concentration of Cu in Sn is 1.67 wt.%^24^. Then the molar heat of transport of Cu atoms in molten Sn at 280 °C was calculated as + 14.65 kJ/mol which is close to that at 250 °C.

Taking temperature gradient (136.50 °C/cm and 154.50 °C/cm) and heat of transport (+11.12 kJ/mol and + 14.65 kJ/mol) into equation (9), the driving force of thermomigration in liquid solder *F*_L_ equals to 4.82 × 10^−19^ N at 250 °C and 6.80 × 10^−19^ N at 280 °C, which is much smaller than those in solid solder as 4.14 × 10^−18^ N^25^ and 1.66 × 10^−18^ N^6^. That is, the growth of IMC at liquid-solid interfaces becomes more sensitive to temperature difference.

### Model of Cu_6_Sn_5_ growth under TM

#### Model of Cu_6_Sn_5_ growth at the hot end under TM

**Figure 9:**
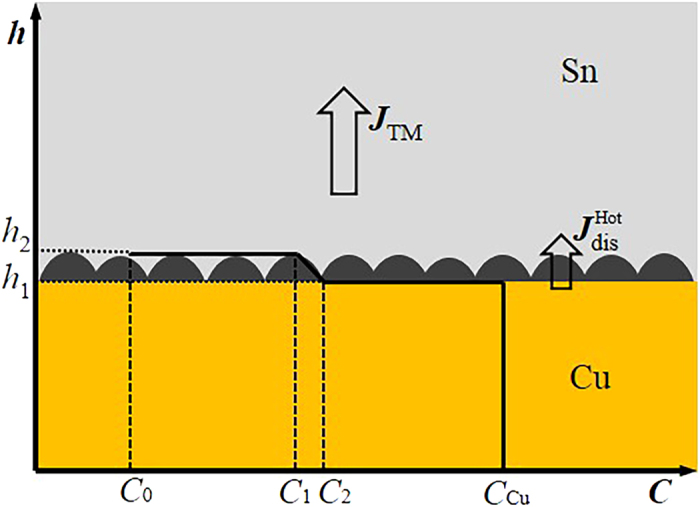
Schematic of Cu concentration profile at the hot end of the Cu/Sn/Cu interconnect undergoing TM.

Figure 9 shows the schematic of Cu concentration profile at the hot end of the Cu/Sn/Cu interconnect undergoing TM. *C*_0_, *C*_1_, *C*_2_, and *C*_Cu_ are the Cu concentrations in solder (near Cu_6_Sn_5_ IMC), solder/Cu_6_Sn_5_ interface, bulk Cu_6_Sn_5_ IMC, and Cu substrate, respectively. *h*_1_ and *h*_2_ indicate the positions of the down and up interfaces of Cu_6_Sn_5_, respectively. The thickness of the Cu_6_Sn_5_ can be obtained through the interface movement of the Cu_6_Sn_5_, which is available by analyzing the flux movement toward and away from the interface. The down interface *h*_1_ and up interface *h*_2_ are moving with velocities of *dh*_1_*/dt* and *dh*_2_/*dt*, respectively. Thus, based on the analysis of Huang *et al.*^3^ and Gan *et al.*^26^, a model was proposed to describe the Cu_6_Sn_5_ growth at the hot end by the following equation


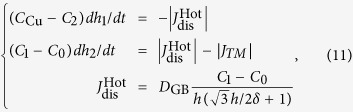


where δ is the average width of IMC grain boundary, *D*_GB_ is the diffusion coefficient of Cu atoms through grain boundary, and *h* = *h*_2_–*h*_1_.

Based on equation (11), the growth rate of Cu_6_Sn_5_ *dh/dt* at the hot end can be expressed as


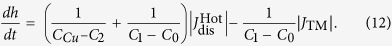


Equation (12) can be further simplified as


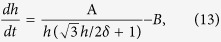


where 
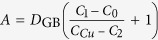
 and 
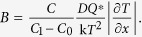


The first term on the right side of equation (13) corresponds to chemical potential gradient and contributes to the parabolic growth law since it has length dependence; while the second term corresponds to temperature gradient and contributes to the linear growth law. When temperature gradient ∂*T*/∂*x* is small or approaches zero, the IMC growth would follow the parabolic law, such as the aging cases. When ∂*T*/∂*x* is large enough for the temperature gradient term to offset the chemical potential gradient term, the IMC would quit to growth, such as the case after TM for 15 min at 250 °C. What’s more, if the temperature gradient term plays a more important role than the chemical potential gradient term under an even larger ∂*T*/∂*x*, decomposition of IMC would occur, such as the case after TM for 30 min at 280 °C.

#### Model of Cu_6_Sn_5_ growth at the cold end under TM

Similarly, the Cu6Sn5 growth at the cold end can be expressed as the following equation


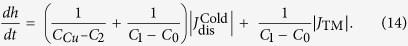


Since Cu atoms can hardly diffuse across the thickening Cu_6_Sn_5_ layer against temperature gradient, the chemical potential gradient term is negligible. The IMC growth only depends on the temperature gradient term and follows a linear growth law.

**Figure 10:**
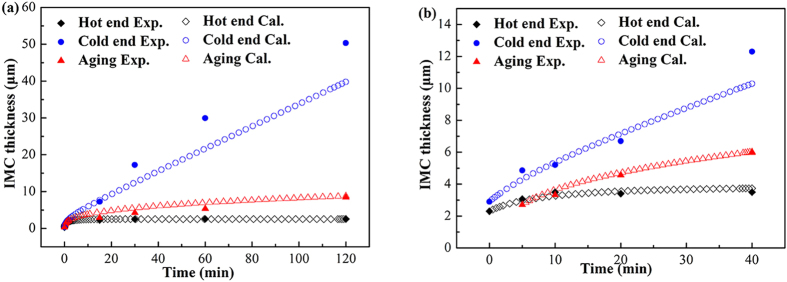
Calculated and experimental results of Cu_6_Sn5 thickness as a function of reaction time: (a) reflowed at 250 °C under temperature gradient of 136.50 °C/cm in the present study; (b) reflowed at 260 °C under temperature gradient of 51 °C/cm in Ref. 11.

A fourth/fifth-order Ronge-Kutta algorithm was applied to numerically integrate equations (13) and (14). Assuming *δ* ≈ 0.05 μm^17^, *C*_1_−*C*_0_ ≈ 0.001^17^, *C*_Cu_−*C*_2_ = 5/11, and *D*_GB_ = 5.5 × 10^−6^ cm^2^/s^27^, the thickness of interfacial Cu_6_Sn_5_ as a function of reaction time were calculated. The aging cases were obtained by setting the temperature gradient as zero. Figure 10(a) shows the calculated and experimental results using the data reflowed at 250 °C in the current study and those for Fig. 10(b) are from Ref. 11. The calculated values are in good agreements with the experimental results, indicating that the proposed model is reasonable.

The thermomigration of Cu atoms results in the significantly asymmetrical growth kinetics of interfacial IMCs at both ends of the interconnections, which may further impact the reliability of devices. Therefore, the consequence in this study suggests that the effect of thermomigration on interfacial IMC growth during soldering reaction is an important consideration in 3D IC packaging technology.

In summary, this study reported the diffusion behavior of Cu and its effect on liquid-solid interfacial reaction in Cu/Sn/Cu interconnects under different temperature gradients. The Cu_6_Sn_5_ and Cu_3_Sn IMCs showed symmetrical growth at both interfaces during isothermal aging and the growth of Cu_6_Sn_5_ was controlled by the volume diffusion of Cu atoms to the Sn/Cu interfaces. During thermomigration, however, asymmetrical growth of interfacial IMCs was clearly observed with Cu_6_Sn_5_ being much thicker at cold end and Cu_3_Sn being hindered especially at hot end. The growth of the Cu_6_Sn_5_ at the cold end followed the linear law with thermomigration time, showing a reaction/thermomigration-controlled growth mechanism; while the Cu_6_Sn_5_ at the hot end hardly grew or even dissolved, showing a grain boundary diffusion/thermomigration-controlled growth mechanism. With simulated temperature gradients of 136.50 °C/cm and 154.50 °C/cm, the molar heat of transport of Cu atoms in molten Sn was calculated as +11.12 kJ/mol at 250 °C and +14.65 kJ/mol at 280 °C, and the corresponding driving force of thermomigration was estimated as 4.82 × 10^−19^ N and 6.80 × 10^−19^ N. A model was proposed to describe the Cu_6_Sn_5_ growth under temperature gradient and the calculated results fitted well with the experimental data from both the present study and the literature.

## Methods

**Figure 11:**
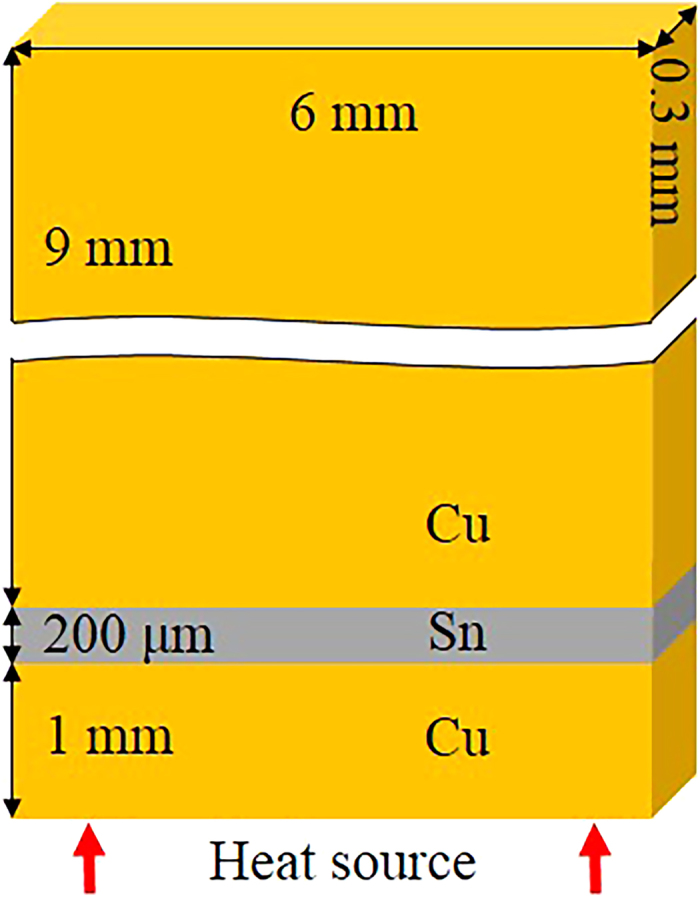
Schematic of a final interconnect.

Cu/Sn/Cu interconnects were prepared by immersion soldering at 250 ± 2 °C for 10 s. Two Cu plates (99.95%) were prepared with one surface being carefully ground, polished, and cleaned in ethanol. After the polished faces were aligned and fixed, the whole configuration was immersed into a pure Sn (99.99%) bath. The gap between the two Cu plates was controlled by two stainless spacers of 200 μm in diameter. After immersion soldering, the specimen was cooled immediately in water to room temperature and then cut into thin interconnects following by grinding and polishing. Figure 11 shows the schematic of a final interconnect. TM experiments were carried out by reflowing the Cu/Sn/Cu interconnects on a hot plate at 250 °C and 280 °C for different durations. Silicone grease with a high thermal conductivity was used to fix the interconnects onto the hot plate. For comparison, isothermal aging experiments were conducted in an Espec STH-120 high temperature chamber at the same temperatures and reaction durations.

The microstructural evolution of the Cu/Sn/Cu interconnects after TM and isothermal aging was examined by scanning electron microscopy (SEM). The composition of the IMC phases and the Cu concentration in the solder layer were identified by energy dispersive X-ray spectroscopy (EDS) and electron probe micro analyzer (EPMA). The area of the interfacial IMC layer was measured using image processing software, and the average thickness was obtained by dividing the area by the line length of the interface. The temperature distribution in the Cu/Sn/Cu interconnects undergoing TM was simulated by finite element method (FEM) using 65 W/m^2^·K as the combined heat transfer coefficient of convection and radiation and 25 °C as the ambient air temperature.

## Additional Information

**How to cite this article**: Zhao, N. *et al.* Growth kinetics of Cu_6_Sn_5_ intermetallic compound at liquid-solid interfaces in Cu/Sn/Cu interconnects under temperature gradient. *Sci. Rep.* **5**, 13491; doi: 10.1038/srep13491 (2015).

## Figures and Tables

**Table 1:** Concentration (at.%) of Cu across the solidified solder layer after reflowed at 250 °C for 15 min and 30 min.

Position	15 min			30 min		
	Cold end	Middle	Hot end	Cold end	Middle	Hot end
1	1.551	2.385	1.969	1.062	1.482	1.168
2	1.428	2.254	1.997	0.993	2.011	1.241

**Table 2:** *K* and *n* values under different conditions.

Temperature	Aging		Hot end		Cold end	
	*K*/(μm/min^n^)	*n*	*K*/(μm/min^n^)	*n*	*K*/(μm/min^n^)	*n*
250 °C	1.15	0.44	1.99	0.06	0.65	0.93
280 °C	1.41	0.45	5.13	−0.16	1.23	0.88
